# Probiotic Modulation of Innate Cell Pathogen Sensing and Signaling Events

**DOI:** 10.3390/nu9101156

**Published:** 2017-10-23

**Authors:** Amy Llewellyn, Andrew Foey

**Affiliations:** 1 School of Biomedical & Healthcare Sciences, Plymouth University Peninsula Schools of Medicine & Dentistry, Drake Circus, Plymouth PL4 8AA, UK; 2 Menzies School of Health Research, John Mathews Building (Building 58), Royal Darwin Hospital Campus, PO Box 41096, Casuarina NT0811, Australia; amy.llewellyn@menzies.edu.au

**Keywords:** probiotics, innate, epithelial cells, dendritic cells, neutrophils, macrophages, signaling, immunomodulation, cytokines, inflammation

## Abstract

There is a growing body of evidence documenting probiotic bacteria to have a beneficial effect to the host through their ability to modulate the mucosal immune system. Many probiotic bacteria can be considered to act as either immune activators or immune suppressors, which have appreciable influence on homeostasis, inflammatory- and suppressive-immunopathology. What is becoming apparent is the ability of these probiotics to modulate innate immune responses via direct or indirect effects on the signaling pathways that drive these activatory or suppressive/tolerogenic mechanisms. This review will focus on the immunomodulatory role of probiotics on signaling pathways in innate immune cells: from positive to negative regulation associated with innate immune cells driving gut mucosal functionality. Research investigations have shown probiotics to modulate innate functionality in many ways including, receptor antagonism, receptor expression, binding to and expression of adaptor proteins, expression of negative regulatory signal molecules, induction of micro-RNAs, endotoxin tolerisation and finally, the secretion of immunomodulatory proteins, lipids and metabolites. The detailed understanding of the immunomodulatory signaling effects of probiotic strains will facilitate strain-specific selective manipulation of innate cell signal mechanisms in the modulation of mucosal adjuvanticity, immune deviation and tolerisation in both healthy subjects and patients with inflammatory and suppressive pathology.

## 1. Introduction

Probiotics have been shown to both augment/modulate homeostatic immune defences and to ameliorate specific infectious, inflammatory and allergic diseases by modulating gut function. Probiotics are described as “Live microorganisms, which, when consumed in adequate amounts, confer a health benefit on the host” by the Food and Agricultural Organization of the United Nations and the World Health Organization [1]. Although probiotics can be beneficial in specific human health and clinical applications, the mechanisms used to modulate immune function and mucosal integrity are poorly understood. In general, there is an ever-increasing body of evidence that describes probiotic bacteria to modulate mucosal immunity at the level of barrier effects, pathogen sensing, innate and adaptive responses. The gut mucosa, the front line barrier to food antigens, pathogens and commensal organisms, is predominated by cells of the innate immune system such as macrophages (MΦs) and dendritic cells (DCs) which, through their ability to produce immune signaling cytokines and act as antigen presenting cells (APCs), can dictate overall immune function as activatory or regulatory. In order to understand how probiotics exert their beneficial effects, the cellular and molecular mechanisms involved in the modulation of innate signaling pathways needs to be clarified. This will be critical for the appropriate selection of probiotic strains for pharmacobiotic strategies used in prophylaxis and the direct treatment of specific clinical conditions.

Probiotics are typically, although not necessarily commensal bacteria. They are generally lactic acid bacteria (LAB), most commonly lactobacilli and bifidobacteria species, although lactococcus, streptococcus and enterococcus species, as well as some non-pathogenic *Escherichia coli* strains are also known probiotics [2]. These probiotics have numerous effects on the gastrointestinal tract (GIT) and the gut-associated lymphoid tissue (GALT) where they modulate intestinal function and immune responses via augmentation of activation (adjuvanticity), and regulation/tolerisation (reviewed in [3]). These effects include the competitive exclusion of pathogens at the intestinal barrier, modulation of dendritic cell (DC) function, influencing T cell polarization and suppression of intestinal inflammation. This results as a consequence of the down-regulation of pro-inflammatory cytokine release from immune cells by the activation, up-regulation of endogenous suppressors, inhibition and cross-regulation of signaling pathways, including nuclear factor-kappa B (NF-κB), mitogen activated protein kinases (MAPK), Janus kinase/Signal transducers and activators of transcription (JAK/STATs) and phosphatidyl inositol 3-kinase (PI3K) [4].

The subtleties of innate immune responses directed by probiotic bacteria are likely to be portrayed in the complexities of signaling pathways activated and the cross-talk between them. Thus, the aim of this review is to explore the many innate immune reception events and signaling pathways modulated by probiotics, and how these affect intestinal function and innate immune responses. The ways in which probiotics are recognised by innate immune receptors will also be explored, with a particular emphasis on pathogen sensing, barrier integrity, anti-microbial and innate immune responses driven by intestinal epithelial cells (IECs) and the immune cells underneath this barrier such as the DCs, MΦs, neutrophils (NΦs) and natural killer cells (NKs).

## 2. Probiotic Modulation of Intestinal Epithelial Cells

**Figure 1:**
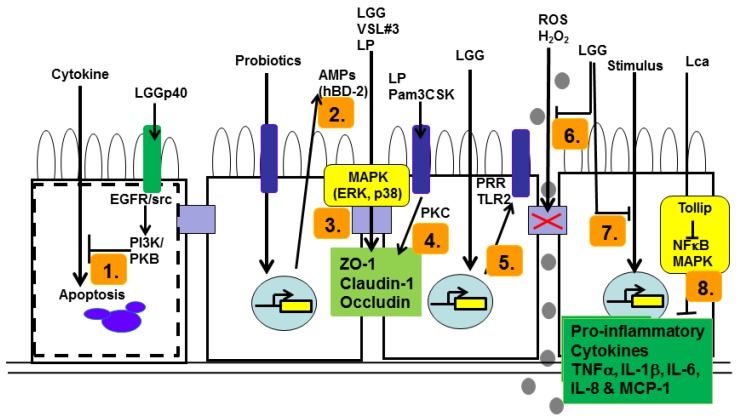
Probiotic modulation of intestinal epithelial cell signaling pathways Probiotic-mediated modulation of epithelial cell responses via activation or suppression of distinct signaling pathways is species-dependent. Mechanisms of modulation are presented above for a range of species of Bifidobacteria, Lactobacilli, Streptococcus, VSL#3 mixture and the yeast *S. cerevisiae*, indicated in the luminal space above the square epithelial cells; additionally, LGG, *L. rhamnosus* strain GG; LGGp40, LGG secreted p40 protein; LP, *L. plantarum*; Lca, *L. casei*. Mechanisms are indicated by numbers 1–8 in orange boxes: 1. Anti-apoptotic, 2. Antimicrobial defence, 3. Reinforcement of tight junction (TJ), 4. Reinforcement of TJ (gene expression & translocation to TJ), 5. Pathogen sensing, 6. Protection against TJ damage, 7. & 8. Suppression of inflammatory cytokines. Arrowed lines are activatory, blunted lines are suppressive/inbibitory. Light blue boxes between epithelial cells are representative of tight junctions. Dark blue boxes represent PRRs, predominantly TLR2. Dark green box on apical surface is an, as yet unidentified receptor for LGGp40. EGFR, Epithelial growth factor receptor; Src, serine-threonine kinase; ZO-1, zonula occludens-1; AMPs, antimicrobial peptides; TNFα, tumour necrosis factor-alpha; PRR, pattern recognition receptors; PKC, protein kinase C; PI3K, phosphatidyl inositol 3-kinase.

In the context of gut homeostasis, there is a fine balance between epithelial cell proliferation, differentiation and apoptosis, allowing this dynamic cellular barrier to continually replace itself, protect from infectious pathogenic agents and to die off prior to cellular transformation resulting from long-term exposure to carcinogenic agents present in intestinal/digesta-associated water. Intestinal epithelial cells (IECs) represent the physical barrier that maintains the segregation between luminal microbes, digesta and the mucosal immune system [5]. Probiotics and commensals can modulate IEC function in a variety of ways, including indirect effects on microbial biofilms [6] and direct effects on IECs via enhancement of barrier function by enhancing tight junctions and mucin production [7]; induction of antimicrobial peptides (AMPs) and heat shock protein production [8,9]; modulation of pro-inflammatory and immunoregulatory cytokines and interference with pathogenesis [10,11]. The functions of the intact epithelial barrier rely on intracellular signaling cascades, on which probiotics exert their effects. These effects are being elucidated by an increasing number of both in vitro and in vivo studies (refer to both Table 1 and Figure 1).

### 2.1. Modulation of Barrier Function

Epithelial growth factor receptor (EGFR) is expressed by IECs and mediates various biological functions, including cellular proliferation, differentiation and cell survival [12]. EGFR is activated by its soluble ligand EGF, which triggers the formation of homo- and hetero-dimers with other ErbB receptor tyrosine kinases, activating the auto-phosphorylation of several cytoplasmic proteins [13]. The tyrosine-phosphorylated EGFR indirectly recruits several adaptor proteins, which activate various downstream signaling pathways such as protein kinase C (PKC), PI3K and MAPK. These pathways induce the production of tight junction proteins and enhance epithelial barrier integrity. Probiotic strains have been described to regulate some of these signaling cascades, augmenting tight junction integrity, survival of IECs and modulation of mucosal barrier immunity [10].

***Enhancement of Tight junction strength:*** Epithelial barrier integrity is determined by the strength of tight junctions (TJ) [12]. These tight junctions are composed of transmembrane proteins [14], whose assembly is dependent on the activation of MAPK signaling pathways. Some of the proteins involved in the formation of tight junctions are zonula occludens (ZO)-1, a scaffold protein; occludin, a transmembrane protein and Claudin-1, an integral membrane protein localized at tight junctions [15]. Probiotics can enhance barrier function by preventing the destruction of intestinal paracellular permeability. It has been shown that *Lactobacillus rhamnosus GG* (LGG) up-regulates the expression of ZO-1, Claudin-1 and Occludin in the human colon-derived intestinal epithelial cell line, Caco-2 [16]. Moreover, the mechanisms by which probiotics control intestinal barrier function are beginning to be clarified. An investigation of the effects of *Lactobacillus plantarum* on Caco-2 intestinal cells induced TLR2 signaling-mediated translocation of ZO-1 to the TJ region between epithelial cells [17]. Pre-treatment of Caco-2 monolayers with *L. plantarum* or the Toll-like receptor-2 (TLR2) agonist, Pam_3_- Cys-SK4 (P3CSK), significantly attenuated the effects of phorbol ester-induced dislocation of ZO-1 and occludin and the associated increase in epithelial permeability. The phorbol ester, PMA, is an analogue of the protein kinase C (PKC)-activating diacyl glycerol (DAG); taken together with the observations above, it is suggestive of a reciprocle relationship between MAPK and PKC signaling. The understanding of this relationship will only be clarified by more detailed studies investigating the relative utilisation of MAPK and PKC isoforms. These effects however, may be strain- and dose-specific. It has also been demonstrated that expression of TLR2 mRNA is up-regulated in IPEC-J2 cells (neonatal porcine mid-jejunum derived) when pre-treated with *LGG*, suggesting that TLR2 recognition of gram-positive probiotic bacteria plays a significant role in strengthening barrier integrity [18]. Furthermore, pre-treatment with *LGG* suppressed the F4^+^ ETEC (Enterotoxigenic *Escherichia coli* K88)-induced increase in the pro-inflammatory cytokine, tumour necrosis factor-alpha (TNFα). These results identify TLR2 stimulation by probiotic bacteria as a regulator of epithelial integrity and may have implications for the understanding of downstream probiotic mechanisms and the subsequent control of intestinal homeostasis (refer to Figure 1).

The downstream effects of TLR activation include two very important signaling cascades, involving the MAPK and NFκB pathways. It was observed that probiotics can modulate intestinal epithelial permeability via up-regulation and activation of MAPK pathways (refer to Table 1). The MAPKs consist of numerous serine-threonine protein kinases of which extracellular signal-related kinases (ERK), c-Jun amino-terminal kinases (JNK), and p38 are the primary members [19]. Activation of ERK and JNK results in activation and nuclear translocation of the transcription factor, AP-1, and the subsequent transcription of pro-inflammatory genes including TNFα. A study carried out by Dai et al. (2012) [20] used the VSL#3 probiotic mixture (consisting of 8 different Gram positive bacteria: *B. longum*, *B. infantis*, *B. breve*, *L. acidophilus*, *L. casei*, *L. delbrueckii* ssp. *bulgaricus*, *L. plantarum* and *Streptococcus salivarius* ssp. *thermophilus*) to characterise the *in vivo* effects on a murine acute dextran sodium sulphate DSS-induced colitis model, and paralleled in vitro TNFα-cultured human HT-29 (human colon adenocarcinoma epithelial) cell effects on tight junction proteins (occludin, ZO-1) and MAPK signaling. It was observed that VSL#3 ameliorated the disease in vivo, due to increased expression of tight junction proteins (occludin and ZO-1) which were dependent on the activation of both ERK and p38 MAPKs. Moreover, the in vitro effects of VSL#3 on the tight junction proteins were abbrogated by SB203580 and U0126, p38 and ERK inhibitors, respectively. These studies are suggestive of probiotic-dependent mechanisms are involved in the regulation of epithelial integrity and that this regulation results from the contribution of both ERK and p38 MAPK signaling pathways to intestinal homeostasis. Of particular interest to this study was the inclusion of investigation focussed on JNK, however the probiotic-mediated barrier repair through the induction of TJ proteins was found to be independent of JNK signaling. Coupled with the understanding of JNK signaling being associated with stress responses; these studies would suggest that there is differential activation of MAPK pathways in reparative versus stress/inflammatory responses in epithelial barriers.

***Preventing apoptosis:*** As indicated earlier, the balance between cell proliferation and cell death represents a fine line between homeostasis, the ability to mount efficient defences to pathogens and mucosal pathology. In addition to tight junction enhancement, probiotics have been shown to confer protection against many cellular stresses, which include oxidative stress-mediated apoptosis [21]. The *LGG* strain ATCC53103 was shown to secrete p40 and p75 soluble proteins in fermented milk, where the p40 soluble protein has been shown to ameliorate cytokine-induced apoptosis in YAMC (young adult mouse colon) cells by the transactivation of EGFR and subsequent activation of the PI3K-downstream substrate, Akt/PKB [21]. It has since been found that p40 stimulates Src (serine-threonine kinase) activation in IECs, which induces the release of EGF via the activation of matrix metalloproteinases (MMPs). Hence, p40 activation of EGFR is src-dependent, as evidenced by Src-inhibition blocking p40-mediated activation of EGFR [22]. This suggests that Src may serve as an upstream mechanism for p40 transactivation of EGFR and the subsequent activation of the downstream signal effector, Akt/PKB in intestinal epithelial cells, which prevents apoptosis. Moreover, in a similar study, *LGG* also attenuated H_2_O_2_-induced disruption of the tight junction complex in IECs [23]. These studies together demonstrate how *LGG* protein products can enhance membrane barrier integrity and protective responses by activation of the anti-apoptotic PKB/Akt in a PI3K-dependent manner to protect IECs from cytokine-induced apoptosis. The ability of probiotics to regulate IEC apoptosis may be a useful strategy for the prevention of reduced membrane integrity caused by enteric infections and inflammatory disorders. The soluble proteins produced by LGG ATCC53103 have been shown to successfully prevent colitis in vivo in a DSS induced mouse model [24]. Just whether this prophylactic protective response can be translated to a therapeutic use in established disease, remains to be clarified (refer to Table 1).

### 2.2. Modulation of Mucosal Immunity

There is increasing research in the use of probiotics for decreasing pathogen-induced pro-inflammatory responses and ameliorating GI diseases. The results from this research may be critical for the evaluation of the mechanisms of action utilised by different probiotic strains and how they can prevent the inflammatory effects of infectious agents (refer to both Table 1 and Figure 1).

Pathogens are recognised by the innate immune systems pattern recognition receptors (PRR). These receptors bind pathogen-associated molecular patterns (PAMPs), which are common conserved structures shared by the vast majority of pathogens. PRRs include Toll-like receptors (TLR), NOD-like receptors (NLR), Rig-1-like receptors (RLRs) and C-type lectins (CLRs). TLRs are membrane bound and provide pathogen surveillance, which upon ligand binding, activate NFκB signaling, leading to the production of pro-inflammatory cytokines, chemokines and antimicrobial peptides [37]. NLRs are cytoplasmic receptors that also provide host defense via the activation of NFκB signaling [38]. To date, the human TLR family consists of 10 members. TLR1, 2, 4, 5, 6, and 10 are cell surface membrane bound and primarily respond to bacterial PAMPs. TLR3, 7 8 and 9 are found on intracellular components, where they respond primarily to nucleic acid-based PAMPs from viruses or bacteria [39]. Interaction of TLRs, except TLR3, with its ligand, leads to the recruitment of intracellular adaptor proteins, which contain a toll-IL-1 receptor (TIR) domain (reviewed in [38]). These include MyD88 (myeloid differentiation primary response gene 88), TIRAP/MAL (TIR domain containing adaptor protein) and TRIF (toll receptor—IL-1 receptor factor). The adaptor proteins interact with the receptor through TIR-TIR binding which results in the recruitment of IL receptor-associated kinases (IRAK-1,2 & -4) and TNF receptor-associated factor 6 (TRAF6). This leads to the activation of the mitogen-activated protein kinases (MAPK) (ERK, JNK and p38), and subsequently transcription factors NFκB and AP-1. Activation of these transcription factors induces production of pro-inflammatory (IL1β, IL-6, IL-8, TNFα) and anti-inflammatory (IL-10) cytokines and anti-viral type 1 interferons (IFNα, IFNβ) [40].

The NFκB pathway is an important signaling cascade for the activation of various immune responses. NFκB is composed of several protein subunits, which regulate the transcription of effector genes including the pro-inflammatory cytokine TNFα and the neutrophil chemokine, IL-8 [41]. During non-stimulatory conditions, NFκB is inactive in the cytoplasm, bound to the inhibitor molecule IκB. Upon activation, IκB is phosphorylated by IKK, which targets it for ubiquitination. NFκB is subsequently freed from IκB, which upon unmasking of the nuclear localization sequence (NLS), the NFκB p65/p50 heterodimer is able to migrate into the nucleus where it functions as a transcription factor at target promoter regions. Among the many upstream signaling proteins involved in NFκB activation is TLR4, which plays a critical role in many intestinal inflammatory diseases [42]. TLR4 recognises LPS from Gram-negative bacteria. Many signaling molecules in the TLR4/NFκB pathway present opportunities for probiotic modulation of activation of pro-inflammatory responses (refer to Figure 1, below). Increasing research efforts have been focused on the down-regulation of the inflammatory response to Enterotoxigenic *Escherichia coli (ETEC)* via modulation of this TLR4/NFκB pathway, thus probiotic bacterial strains would appear to modulate LPS/Gram-negative bacteria-induced inflammatory responses.

**Figure 2:**
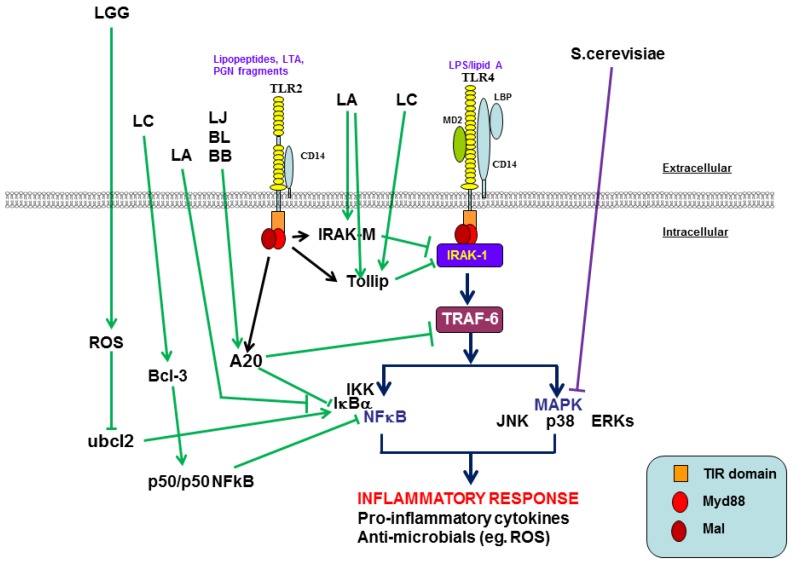
Probiotic modulation of pathogen sensing by TLR-2 and TLR-4 signaling pathways. Probiotic-mediated modulation of TLR-2 and TLR-4 signaling via direct and indirect mechanisms of activation or suppression of distinct signaling molecules/pathways is strain-dependent. Mechanisms of modulation are presented above for a range of strains of bifidobacteria (BL = *B. longum* strain BB536; BB = *B. breve* strain M-16V), lactobacilli (LGG = *L. rhamnosus* strain GG; LC = *L. casei* strain 0LL2768; LJ = *L. jensenii* strain TL2937; LA = *L. amylovorus* strain DSM16698T), and the yeast *S. cerevisiae*, indicated in the extracellular space above the intracellular post-membrane receptor signaling pathways. TLR signal transduction is initiated by TLR signal adaptor molecules, represented by red ovals, which bind the TIR domains (orange box) on the TLR cytoplasmic region. Signal is passed downstream via activation of IRAK-1 (purple box) and TRAF-6 (maroon box), resulting in the activation of NFkB and MAPK pathways. Arrowed lines are activatory, blunted lines are suppressive/inhibitory. Probiotic bacteria regulate inflammatory responses via induction of a range of endogenous negative regulators of TLR signaling (IRAK-M, Tollip, A20, Ubcl2, Bcl-3 and p50/p50 NFkB homodimer). TLR2 ligation may induce a suppressive effect on TLR4-mediated inflammatory responses via expression/activation of IRAK-M and Tollip. *S. cerevisiae* can exert a suppressive effect on inflammatory responses via inhibition of MAPK pathways (p38, JNK and ERKs). At this time, studies represented in this figure use a range of cells/cell lines; selective manipulation of signal checkpoint molecules and pathways is likely to represent a cell and cell subset-specific nature.

***Negative regulation of TLR4 signaling:*** TLR4 is expressed on epithelial and immune cells and responds to PAMPs on Gram-negative bacteria, most commonly LPS. The intestinal immune system therefore, must constantly maintain a tightly controlled balance between activation and inhibition of TLRs to avoid detrimental unintended stimulation by the microflora and inappropriate inflammatory responses. In order to prevent inappropriate responses to commensal and pathogenic bacteria, various negative regulators exist to attenuate TLR signaling [43] (refer to Figure 2). Negative regulators include membrane bound suppressors such as single immunoglobulin interleukin-1-related receptor (SIGIRR), and TNF-related apoptosis-inducing ligand receptor (TRAILR) and intracellular inhibitors, which can inhibit TLR signaling at multiple levels. The intracellular inhibitors include sMyD88 (short isoform), interleukin-1 receptor-associated kinase M (IRAK-M), suppressor of cytokine signaling 1 (SOCS1), phosphatidylinositol 3-kinase (PI3-K), Toll interacting protein (Tollip), and A20. Negative regulators ensure chronic inflammatory TLR responses to MAMPs (microbial associated molecular patterns) from commensal bacteria do not occur. In fact, it is being discovered that many probiotics can modulate TLR negative regulators in IECs to inhibit pathogen-induced inflammation and possibly contribute to the tolerance of the intestinal barrier to commensal bacteria [44].

*Lactobacillus casei* OLL2768 has been shown to attenuate the ETEC-induced pro-inflammatory response by inhibiting NFκB and p38 MAPK signaling pathways in bovine intestinal epithelial (BIE) cells, which reduced the expression of IL-6, IL-8, IL-1β and MCP-1 [28]. This was associated with the negative regulation of TLR4 signaling via the up-regulation of Toll interacting protein (Tollip), which inhibits the TLR adaptor protein, IRAK subsequently preventing the over-expression of NFκB and therefore inflammatory damage. In addition, *L. casei* OLL2768 also up-regulated the nuclear protein, B-cell lymphoma 3- encoded protein (Bcl-3). Bcl-3 is a member of the NFκB family, which is able to stabilize repressive NFκB homodimers, for example p50/p50. The activation of Bcl-3 can therefore prevent the binding of transcriptionally active dimers to gene promoters and effectively inhibit pro-inflammatory responses. Another lactobacilli strain isolated from the intestines of unweaned pigs has shown anti-inflammatory effects in human Caco-2/TC7 cells. *L. amylovorus* DSM 16698T protects IECs against the pro-inflammatory response triggered by ETEC K88 through the repression of the pro-inflammatory cytokines IL-1β and IL-8 [27]. The protective activity of *L. amylovorus* DSM 16698T was exerted by inhibiting various steps of TLR4 signaling via the modulation of negative regulators. *L. amylovorus* inhibits the ETEC induced activation of TLR4 and MyD88, and the phosphorylation of IKKα, IKKβ, IκBα and NFκB subunit p65, which subsequently inhibited the over-production of inflammatory cytokines IL-8 and IL-1β. These anti-inflammatory effects are modulated by the activation of the negative regulators of TLR4 signaling, Tollip and IRAK-M. Moreover, the use of anti-TLR2 neutralising antibodies has helped clarify that the probiotic-dependent suppression of TLR4 signaling was dependent on TLR2. Other lactobacilli strains have also demonstrated anti-inflammatory effects via the activation of negative regulation of TLR signaling. *Lactobacillus jensenii* TL2937 down-regulates TLR4-dependent NFκB and MAPK activation, consequently decreasing the expression of pro-inflammatory cytokines and chemokines caused by ETEC or LPS challenge [32]. Furthermore, this down-regulation was shown to be associated with the up-regulation of three negative regulators of TLR4 signaling; A20, Bcl-3 and mitogen-activated protein kinase 1 (MKP)-1. A20 is an ubiquitin editing protein, which inhibits activation of NFκB [45]. This protein functions to target proteins for proteasomal degradation via the attachment of polyubiquitin chains to target proteins. Tumour necrosis factor (TNF)-α-receptor-associated factor 6 (TRAF6) is a common target for A20 and therefore facilitates the down-regulation of NFκB signaling in response to TLR activation [46]. In a similar study to Shimazu et al. (2012) [32], the potential immunomodulatory effects of bifidobacteria strains were investigated [25]. *Bifidobacterium longum* BB536 and *B. breve* M-16V strains were found to significantly suppress IL-8, MCP-1 and IL-6 levels in porcine intestinal epithelial (PIE) cells in response to ETEC PAMPs. These anti-inflammatory effects were shown to be associated with the modulation of NFκB and MAPK pathways via the up-regulation of the ubiquitin-editing enzyme, A20. Moreover, the activity of A20 was abolished when anti-TLR2 blocking antibodies were used. This suggests that bifidobacteria may induce cross-tolerance in IECs through their interaction with TLR2 and the up-regulation of A20. It may be that certain probiotics share a common mechanism for their immunomodulatory effects as it has also been demonstrated that *L. amylovorus* activates the negative regulators of TLR4 signaling in a TLR2-dependent manner. These studies demonstrate potential therapeutic strategies for the treatment of intestinal inflammatory disorders in humans, induction of negative regulators of signaling cascades may be crucial mechanisms whereby probiotics exert anti-inflammatory effects.

***Modulation of pro-inflammatory cytokines:*** Epithelial barrier integrity can be affected by pro-inflammatory cytokines, which are induced by activation of signaling pathways such as NFκB and MAPK. Many studies have identified probiotic strains that suppress the production of pro-inflammatory cytokines from IECs, via modulation of many checkpoints in these signaling pathways, therefore reducing the detrimental inflammatory damage to the intestinal epithelial barrier caused by pathogen-induced inflammation. Studies have shown that pretreatment of Caco-2BBe (brush border-cytoskeletal model) cells with LGG decreased TNFα induced NFκB activation due to inhibition of nuclear translocation of the NFκB subunit p65 [47] (refer to Figure 1). Subsequent gene expression studies demonstrated that the chemokine CXCL-8 (IL-8, chemotactic for neutrophils) and CCL-11 (Eotaxin, chemotactic for eosinophils) protein levels were decreased in LGG-treated, cytokine-challenged cells. Moreover, LGG inoculation prevented TNFα-induced ZO-1 disruption. These findings indicate that LGG alleviates the effects of pro-inflammatory cytokine induced epithelial barrier disruption and further pro-inflammatory cytokine production through inhibition of NFκB signaling. *B. lactis* has been shown to significantly suppress NFκB activation in human HT-29 cells, when stimulated with the pro-inflammatory cytokines IL-1β and TNFα and LPS [26]. The down-regulation of activated NFκB correlated with the reduction of NFκB-binding activity and suppression of IκB degradation. *L. reuteri* inhibits intestinal LPS-induced phospho-IκB activity in newborn rats with necrotising enterocolitis (NEC) in ex vivo experiments [33]. This paralleled down-regulation of mRNA expression of IL-6, TNFα, and IL-1β. Furthermore, intestinal protein levels of TLR4 were also significantly reduced. This study demonstrates probiotic ability to down-regulate NFκB activity, which corresponds to reduced production of inflammatory mediators but can also reduce the expression of TLR proteins hence regulate pathogen sensing.

Another way by which IκB degradation can be suppressed, is through increased amounts of ROS. Studies have recently identified that probiotics modulate inflammatory responses by inducing local generation of reactive oxygen species (ROS) [48]. ROS can regulate cellular processes through oxidative inactivation of key regulatory enzymes. Specifically, *LGG* can induce ROS generation in intestinal epithelia, which are able to exhibit increased oxidation of the Ubc12 enzyme [30]. Ubc12 is responsible for the ubiquitination of the inhibitory molecule IκB, therefore IκBα is not targeted for proteasomal degradation. Thus, NFκB remains bound to IκBα in the cytosol, unable to function as a transcription factor and inducing gene expression. Furthermore, *LGG* was able to prevent intraperitoneal TNFα-induced intestinal activation of NFκB in an ex vivo model of distal small intestines isolated from immature C57BL/6J mice. This indicates that *LGG* reduces inflammatory signaling by inducing ROS generation. Modulation of inflammatory signaling by production of endogenous signals presents a mechanism for reducing pro-inflammatory responses to non-pathogenic stimuli and may have implications for preventing inflammatory bowel diseases (IBD).

Recently, the commensal strain *Streptococcus salivarius* K12 was shown to suppress NFκB activation, correlating with decreased IL-8 production [35]. The conditioned supernatant of *S. salivarius* inhibited NFκB activity in HT-29 cells in response to TNFα, suggesting that the anti-inflammatory properties on IECs are induced by an active metabolite from *S. salivarius*. Furthermore, this supernatant inhibited NFκB activity induced by a variety of stimuli including IL-1β and the TLR5 ligand, flagellin. Therefore, it was hypothesized that the inhibitory compound is localized downstream of the receptors which is common to TNFα, IL-1β and flagellin receptors. This indicates that *S. salivarius* may be involved in molecular cross-talk with a variety of receptors and may have potential for modulating the hosts mucosal immune response in inflammatory disorders.

Probiotics can also affect MAPK signaling pathways to modulate cytokine production. Suppression of expression of the pro-inflammatory cytokines IL-6 and IL-8 has been associated with the decreased phosphorylation of ERK1/2 and p38. This was observed in IECs treated with *Saccharomyces cerevisiae* (*Sc*). It was shown that IPEC-1 cells inhibited the ETEC-induced pro-inflammatory response when pretreated with *Sc* [34]. This inhibition was associated with the decrease of ERK1/2 and p38 MAPK phosphorylation, suggesting that *Sc* can inhibit ETEC-induced inflammation at the epithelial barrier.

Numerous studies have shown that certain strains of probiotics modulate the production of pro-inflammatory cytokines. However, another way by which probiotics may exert immunomodulatory effects is by stimulating the production of the anti-inflammatory cytokine IL-10. In one such study, gene expression changes were observed in healthy 2-week old mouse colon samples 6 hours post oral ingestion of *LGG.* In vivo examination of gene expression changes demonstrated that *LGG* down-regulated the expression of TNFα and MIP-2, but failed to alter IL-10. However, *LGG* did induce mRNA expression of the IL-10R2 subunit of the IL-10 receptor [29]. IL-10 initiates an anti-inflammatory response by binding to its receptor, which activates the JAK1/STAT3 pathway, where STAT3 is phosphorylated [49]. Activated STAT3 inhibits the expression of pro-inflammatory genes such as TNFα and MIP-2 and up-regulates the gene expression of members of the suppressor of cytokine synthesis (SOCS) family. In the colons harvested from *LGG*-treated mice, there was a significant increase in activated phospho-STAT3 and SOCS-3 expression, which correlated with decreased expression of MIP-2 and TNFα. This study demonstrated the *LGG* anti-inflammatory effects are mediated by STAT3 activation and downstream SOCS3 production and is most likely dependent on induction of IL-10R2 expression and the consequent signaling through this receptor chain upon ligation of the anti-inflammatory cytokine, IL-10.

### 2.3. Probiotic Modulation of IEC TLR Expression Regulates Mucosal Intestinal Immunity and Tolerance

IECs regulate translocation of luminal antigens and microorganisms into the lamina propria and have been described to suppress DC activation and contribute to tolerance induction [50]. DCs are present in the Peyer’s patches and recgonise antigens present in the lumen, which have been transported to the Peyer’s patch via specialised IECs called microfold- or M cells [51]. DCs can also protrude between IECs and sample antigens in the intestinal lumen [52]. Upon antigen recognition, DCs transport the antigen within the patch or to the mesenteric lymph nodes where they are presented to lymphocytes. It is probable that DCs can influence the outcome of T cell activation and induce regulatory T cell populations that are essential for mucosal tolerance. Thus, PAMP/antigen capture and recognition is fundamental to IEC/DC determination of immune activation or tolerisation.

***Activation of apical TLR9 by probiotics:*** Under inflammatory conditions, IECs express increased TLR2 and TLR4 on the apical surface, and this is associated with inflammatory bowel disease [53]. In contrast, apically-derived TLR9 stimulation has been described to be anti-inflammatory. It has been shown that basolaterally-derived TLR9 activation in IECs, signals IκBα degradation and activation of NFκB, whereas apically-derived TLR9 stimulation induces a potentially anti-inflammatory/regulatory response in which ubiquitinated IκB accumulates in the cytoplasm preventing NFκB activation [54]. This suggests that apical but not basolateral TLR expression and activation in IECs can maintain colonic homeostasis and regulate tolerance and inflammation.

TLR9 recognises unmethylated CpG motifs of bacterial DNA, which activates many downstream signal pathways including NFκB. A study has demonstrated that living probiotics, probiotic DNA and the synthetic oligodeoxynucleotides containing CpG motifs from LGG and *B. longum* BB536, increased the levels of TLR9 mRNA and NFκB, as well as IκBα phosphorylation compared to a non-CpG control group in an OVA-induced food hypersensitivity mouse model [55]. This correlated with skewing towards a Th_1_ immune response, and increased percentage of CD4^+^CD25^+^ Treg cells (regulatory T cells). This data suggests that activation of TLR9 by the combination of living probiotics or probiotic DNA can prevent allergic responses by immune modulation. In line with this study, apical exposure of polarized HT-29 to DNA from *LGG* was found to attenuate TNFα enhanced NFκB activity by reducing IκBα degradation and p38 MAPK phosphorylation [56]. This anti-inflammatory effect of *LGG* was indeed, mediated by TLR9 signaling; the silencing of TLR9 abolished the inhibitory effect of *LGG*.

A recent study has postulated that the capacity of a probiotic to stimulate immune responses is species-specific and correlates with the frequency of motifs known to exert immunosuppressive functions [57]. The study found that *Lactobacillus paracasei* DNA was enriched in suppressive sequences and these correlated with the probiotic capacity to exert immunosuppressive functions in Lamina propria DCs (LpDC). In particular, *L paracasei* DNA was able to inhibit DC activation and induce CD4^+^ Foxp3^−^ Treg cells in a dose-dependent manner. These findings support the evidence that probiotics exert their immunomodulatory effects by stimulation of TLR9, however these are species and dose specific. Further, this suggests that a balance between regulatory and stimulatory motifs exists to induce gut immune homeostasis, demonstrating that these regulatory motifs enriched in probiotics, targeting TLR9, could be exploited for therapeutic purposes.

## 3. Probiotic Modulation of DCs

### 3.1. Activation of DCs

IECs are important in driving the development of tolerance by suppressing DC activation, which subsequently controls the suppressive function of T regulatory cells (Tregs) [58]. IECs have also been described to induce the development of CD103^+^ DC. This population of tolerogenic DCs induces Foxp3^+^ Treg cells and is dependent on TGFβ and the dietary metabolite, retinoic acid (RA) [59], both being produced by IECs. It is unknown whether TLR activation of IECs influences the generation of tolerogenic DCs. However, it is clear that TLR stimulation in the intestinal epithelium plays an important role in regulating mucosal immunity.

In one particular study, *Bifidobacterium breve* was shown to induce the development of IL-10-producing Tr1 cells [60]. Intestinal CD103^+^ DCs mediated the induction of *B. breve* dependent Tr1 cells. It was shown, using intestinal CD103^+^ DCs from *MyD88 ^−/−^* and *TLR ^−/−^* mice, that the tolerogenic DCs were activated via TLR2/MyD88-dependant production of IL-10 and the Th_2_-inducing IL-12 family member cytokine, IL-27. These findings demonstrate that *B. breve* can prevent intestinal inflammation through induction of IL-10-producing Tr1 cells, mediated through TLR2/MyD88 signaling. Other studies have demonstrated the anti-inflammatory effects of probiotics are CD103-dependent. However, these effects are not dependent on the same PRR and therefore demonstrate that the anti-inflammatory effects are strain-specific. *Lactobacillus salivarius* Ls33 induced the development of CD103^+^ DCs and CD4^+^ Foxp3^+^ regulatory T cells in an IL-10-dependent manner [61]. Purification of PGN from Ls33 demonstrated that the protective effects of *L. salivarius* were NOD2-dependent but MyD88-independent. Another study has identified a probiotic mixture, IRT5 (*Streptococcus thermophilus*, *L. reuteri*, *B. bifidium*, *L. acidophilus* and *L. casei*), which up-regulates the generation of CD4^+^Foxp3^+^ regulatory T cells (Tregs) from the CD4^+^CD25^−^ population [62]. This conversion of T cells into Tregs is mediated by regulatory DCs, which express IL-10, TGFβ, COX-2 and tolerance-associated enzyme, indoleamine 2,3-dioxygenase (IDO), which regulates T cell function through the depletion of tryptophan. Administration of this probiotic mixture had therapeutic effects in experimental inflammatory bowel disease, which was associated with enrichment of CD4^+^Foxp3^+^ T regs in the inflamed regions and subsequently down-regulation of Th_1_, Th_2_, and Th_17_ cytokines without apoptosis. These results represent an applicable treatment of inflammatory immune disorders, which enhance the generation of regulatory DCs and Tregs (refer to Table 2).

The diversity of the gut microbiota influences the gut immune system and plays a major role in inflammatory bowel diseases and allergies. These pathologies are highly stimulated by helper T cell (Th) cell-skewing, induced by strong stimulatory bacteria in the gut microbiota. Lactobacilli and bifidobacteria can have a positive effect on these inflammatory responses. Zeuthen et al. [63] reported that beneficial effects of bifidobacteria are mediated by TLR2 recognition. The combination of *L. acidophilus* X37, *L. paracasei* Z11, *L. casei* CRL431, LGG, *B. longum* Q46, *B. bifidum* Z9, *B. breve* 20091, and *B. bifidum* 20082a decreased IL-12 and TNFα and increased IL-10 levels, which inhibited the Th_1_ skewing effect induced by strong immunostimulatory lactobacilli. It was shown that the immunostimulatory effect of bifidobacteria is TLR2-dependent and NOD2-independent, suggesting that bifidobacteria act as immunoregulators through interaction of lipoprotein with TLR2. Furthermore, probiotics and their supernatants have immunomodulatory effects in human intestinal-like DCs, mediated by cytokines and TLR recognition of MAMPs. *Bifidobacterium breve CNCM-I-4035* and its cell-free culture supernatant (CFS) have been shown to decrease pro-inflammatory cytokines and chemokines in DC when challenged with *Salmonella enterica* serovar *Typhi* CECT725, which was mediated by increased expression of TLR9 and TLR5 [64]. In contrast, the live strain *B. breve CNCM-I-4035* was a potent inducer of pro-inflammatory cytokines including TNFα, IL-8 (CXCL8, recruitment of neutrophils, basophils and Tc) and RANTES (CCL5, recruitment of memory T cells, monocytes, NK cells and immature DCs). Moreover, the CFS increased the expression of Caspase 8, Tollip and IRAK4, whereas these genes were down-regulated by the live bacteria. This suggests that the anti-inflammatory effects of the CFS may be mediated by inhibition of TLR signaling as Tollip is an adaptor molecule which binds TLR2 and TLR4 to inhibit MyD88 binding and activation, therefore inhibiting TLR signaling and subsequently pro-inflammatory cytokine production [65]. However, this story is further complexed by the recognition that IRAK4 is able to bind and phosphorylate Tollip, preventing its ability to interact with the TLR pathway. The supernatants of various other probiotic strains have been investigated for their immunostimulatory effects. The CFS from *Lactobacillus paracasei* CNCM I-4034 and *Lactobacillus rhamnosus CNCM I-4036* decreased pro-inflammatory cytokines and chemokines in human intestinal DCs challenged with *Salmonella enterica* serovar *Typhi* CECT725 and *Escherichia coli* [66,67].

Finally, an ex vivo investigation, using murine bone marrow-derived DCs, has demonstrated that there is a level of cross-regulation/cross-talk between species of probiotic bacteria, hence fine-tuning immune signaling in DCs. *Lactobacillus* spp displayed differential induction of IL-12 and TNFα, whereas all strains tested augmented expression of the DC maturation markers, MHC II and CD86. When investigating two probiotic strains, *L. reuteri* DSM12246 (poor IL-12 inducer) and *L. casei* CHCC3139 (high IL-12 inducer), it was observed that *L. reuteri* strain inhibited IL-12, IL-6 and TNFα induction and reduced CD86 up-regulation by the *L. casei* strain [68]. Thus, cross-talk between probiotic species also has a role to play in defining the overall probiotic response as immune-activatory/pro-inflammatory or immunosuppressive to DCs.

In summary, a specific probiotic bacterial strain could regulate gut homeostasis by facilitating induction of Treg cells (refer to Table 2), inhibiting T cell-mediated mucosal inflammation, increasing production of anti-inflammatory cytokines or decreasing pro-inflammatory cytokines, and mediating TLR signaling. The combinations of different probiotics for treatment of IBD should be researched further, as various combinations have shown promise in reducing inflammation in experimental models. The use of CFS from specific probiotic strains may also prove successful for fine-tuning immunomodulatory effects in vivo.

### 3.2. Probiotic Modulation of NK Cells via Cross-Talk with DCs

Natural killer cells (NKs) play a crucial role in the immune response to tumours and viruses. NKs can distinguish between normal healthy cells and abnormal cells with altered or missing MHC class I molecules [69]. Upon recognition of the abnormal cells, NKs can elicit the secretion of immune mediators including IFNγ and TNFα or direct cytolysis of the infected or transformed cells. In addition, NKs cells can be indirectly activated by secreted soluble factors from DCs such as IL-12, IL-18 and type 1 interferons (IFNα, IFNβ). The secretion of cytokines by DCs and indeed MΦs depends on the nature of the stimuli received from microbial products. Recent studies have identified that LAB-induced DC regulation may affect NK cell activity and subsequent anti-tumour or anti-viral immune responses.

NK cell activity has been shown to be enhanced by LAB in healthy adults. Administration of *Lactobacillus casei* Shirota (*LcS*) to patients whose colonic polyps had been surgically removed significantly reduced the recurrence of colorectal cancer. The specific mechanisms of action of LcS on NK cell activity have also been explored. LcS induced IL-12 and TNFα production, which positively correlated with NK activity [70]. Other studies have revealed that exposure of human DCs to LAB induces activation, proliferation and cytotoxicity in NK cells and subsequent NK-derived IFNγ secretion [71]. This suggests that LAB modulate IFNγ production in NK cells in a DC-dependent manner. However, not all probiotics have the same IFNγ-inducing capability. One study identified that only DCs matured by *L. acidophilus* induced secretion of IFNγ from NK cells [71]. Combining *L. acidophilus* with a non-inducing LAB completely abrogated DC-mediated IFNγ production by NK cells. The mechanisms involved in DC-IL-12 production by *L. acidophilus* have still not been completely elucidated. However, this evidence suggests that combinatory probiotic therapy maybe counter-productive with respect to NK-mediated immunity. In addition, the specific recognition of particular LABs by DCs and NK cells are slowly being uncovered. It was found that oral intake of *L. pentosus* S-PT84 in C57BL/6 mice significantly enhanced NK activity, this increased activity correlated with activation of IL-12 production by CD11c^+^ DCs in a TLR2/TLR4-dependant manner [72].

The main action LABs have on NK cells is the enhancement of their cytotoxic activity and therefore anti-tumour responses rather than the increase in NK cell count. In agreement with this, several human studies have found that LcS supplementation enhanced NK cell activity where in vivo studies revealed this to be due to IL-12 [73,74]. This evidence together suggests that some LAB efficiently initiate NK/DC interactions, which subsequently increases the cytolytic potential of NK cells in mucosal immunity. The potential of LAB strains to differentially induce IFNγ production by NK cells highlights the ability to use these probiotics to modulate cytokine balance and to promote anti-tumour cytotoxic immune responses. This demonstrates the potential to modulate immune dysregulation associated with specific allergies and diseases where Th_1_ cell polarisation is the cause of the pro-inflammatory reaction including the ability to stimulate anti-tumour responses.

With regards the signaling modulated by probiotic microbes, little direct evidence exists. What can be proposed is that, due to NK cell reliance on activation by IL-12, IL-18, the relative balance between KIR/KAR ligation and pathways specific to induction, expression and secretion of IFNγ, NK signaling may be modulated by probiotic bacteria at many levels.

## 4. Probiotic Modulation of Neutrophils

Neutrophils are fundamental to innate immunity and recognition/responsiveness to pathogenic challenge. They are short-lived myeloid cells that readily respond to infection via the oxidative-dependent production of toxic reactive oxygen species (ROS), referred to as oxidative burst, and by phagocytic clearance; both facilitated by neutrophil extracellular traps (NETs). Any such effects of probiotic bacteria on neutrophils is likely to target its effector functions: ROS generation, phagocytosis, NET formation, hydrolytic enzyme activity, chemokine-mediated recruitment and inflammatory cytokine secretion; thus having a profound effect on neutrophil-mediated responses associated with acute infection and chronic infection/immunopathology (refer to Table 3 below).

To date, little is known how probiotic bacteria influence signaling pathways involved in neutrophil effector functionality. What is known is by inference of modulation of effector molecules, which are typically regulated by established signal pathways. *B. animalis* MB5 and *L. rhamnosus* GG strains have been shown to suppress enterotoxigenic *E. coli* K88 induction of the NFκB-dependent inflammatory mediators TNFα, IL-1β, IL-8, Gro-α and ENAP-78 [76], resulting in a corresponding inhibition of neutrophil transmigration. In contrast however, *L. rhamnosus* NutRes1 increased the distal colon expression of CCL2, TNFα, IL-1β, IL-6 and the pathogen-sensing receptors, TLR2/6 whereas neutrophil numbers (Ly-6B.2+) and activity (MPO) were reduced in a murine DSS-induced chronic colitis model [79]. *L. rhamnosus* GG has also been shown to inhibit NET formation and as a consequence, reduced ROS production and prevention of tissue destruction through chronic inflammation [84]; the suppression of ROS production being suggestive of an inhibitory effect on NFκB. Additionally, the cell-wall extract of *L. gasseri* ATC33323 upregulated the expression of TNFα, IL-1β, MIP-1α and MCP-1 in a Sprague-Dawley rat model of sepsis [82], whereas conditioned medium from *L. rhamnosus* L34 and *L. casei* L39 differentially regulated NFκB and c-Jun activation in a *C. difficile*-induced colitis model: L34 suppressed NFκB and L39 suppressed both NFκB and c-Jun involved in IL-8 production, hence neutrophil activation and recruitment [81]. Finally, granulopoeisis or the de novo generation of neutrophils is dependent on G-CSF, which induces the expression of neutrophil-specific genes in a STAT3/STAT5-dependent manner, negatively regulated upon the expression of SOCS3. Observations that specific LABs induce G-CSF, STAT3 activation and SOCS3 are suggestive of a distinct role for probiotics in controlling neutrophil numbers and activity through a cytokine receptor-JAK/STAT-SOCS axis [89,90,91] and (reviewed in [92]). Thus, probiotic modulation of neutrophil signaling is in its infancy and can be summarised to both positively and negatively regulate NFκB, JAK-STATs and JNK MAPK signaling in a manner that is determined by probiotic strain, probiotic format (whole bacteria, cell-wall extract or secreted molecules), PRRs utilised and context of neutrophil/tissue model (homeostatic or pathological) being investigated.

## 5. Probiotic Modulation of Macrophage Signaling Pathways Influences Cytokine Production

Probiotics modulate various signaling pathways within macrophages and have subsequent immunoregulatory functions on mucosal immunity. Extensive research has focused on the modulation of cytokine production by probiotics. In addition to the innate pro-inflammatory cytokines such as TNFα, IL-1β, IL-6 and IL-8, particular attention has been focussed on IL-10 and IL-12, as the production of these cytokines by macrophages and other immune cells in response to microbes can determine the type of immune response downstream of innate sensing. As in IECs, various signaling pathways are the target of probiotic modulation, including NFκB and ERK1/2, p38 and JNK MAPKs. The mechanisms by which the production of cytokine secretion is modulated by probiotics are beginning to be uncovered (refer to Table 4).

### 5.1. Modulation of Pro-Inflammatory Cytokines

Probiotics can suppress inflammation by inhibiting various signaling pathway checkpoints. These signaling pathways include NFκB pathway, which may also be associated with changes in MAPK pathways and PRRs (refer to Table 4). Various probiotics can inhibit IκBα phosphorylation or ubiquitination and the subsequent degradation of this NFκB inhibitor [93], and decrease p65 nuclear translocation resulting in reduced NFκB DNA binding [94]. Select probiotics can also inhibit LPS binding to the CD14 receptor, resulting in an overall reduction in NFκB activation and therefore pro-inflammatory cytokine production [95]. Some of these probiotics can also inhibit MAPK pathway checkpoints, indicating that both NFκB and MAPKs play a role in pro-inflammatory cytokine production and that the use of probiotics, targeting these pathways may have a profound anti-inflammatory effect. For example, LTA (lipoteichoic acid, a TLR2 ligand) isolated from *Lactobacillus plantarum* (pLTA) inhibited the LPS-induced (TLR4-specific) TNFα production by decreasing the degradation of IκBα and IκBβ resulting in suppression of NFκB activation [96]. Additionally, pLTA pretreatment inhibited the phosphorylation of ERK, JNK and p38 MAPKs in THP-1 monocytic cells, which is suggestive of signaling pathways modulated by endotoxin tolerisation (ET). Upstream of the NFκB and MAPK pathways is the recognition of microbes by PRRs, and these components are also affected by pLTA. The LPS-induced expression of TLR4, NOD1 and NOD2 is suppressed by pLTA, along with the induction of IRAK-M, which acts as a negative regulator of TLR signaling [96]. This suggests that pLTA induces tolerance to LPS (ET), since these PRRs are involved in LPS recognition. However, expression of the LPS co-receptor, CD14, was increased in pLTA tolerised cells, suggesting that CD14 also participates in the interaction between TLR2 and LTA, which may result in decreased interaction between CD14 and LPS, therefore reducing inflammation (refer to Table 4). These results suggest that pLTA may be effective in the prevention and treatment of LPS-induced septic shock. Furthermore, purified pLTA inhibits *S. aureus* LTA (aLTA)-induced TNFα production in THP-1 cells, thus exhibiting a level of TLR2-mediated homo-tolerisation [97]. The effects of pLTA on NOD signaling have also been further investigated. The pro-inflammatory response induced by *Shigella flexneri* PGN (flexPGN) on THP-1 cells was reduced, upon pretreatment with pLTA: resulting in a significant reduction in TNFα and IL-1β production, which was associated with down-regulation of NOD2 expression, suggesting that pLTA can modulate flexPGN-induced inflammation [98]. Furthermore, pLTA–tolerant THP-1 cells had reduced phosphorylation of ERK, JNK and p38 MAPKs as well as decreased NFκB activity. These results indicated that pLTA could induce cross-tolerance between TLR2 and NOD2 signaling against a NOD2 agonist such as flexPGN (refer to Figure 2). (For further information on probiotic modulation on ET, refer to Section 5.6).

In addition to modulation of the NFκB pathways, probiotics can inhibit the transcription factor AP-1 (heterodimer of fos/jun) via inhibition of the MAPK-regulated c-Jun. A specific strain of *L. reuteri,* ATCC PTA 6475, suppressed TNFα transcription by inhibiting activation of c-Jun and subsequently AP-1 [99] (refer to Figure 2). The levels of pro-inflammatory cytokines can also be modulated by activation of suppressor of cytokine signaling (SOCS) family proteins. SOCS proteins are negative regulators of cytokine signaling pathways mediated by JAK-dependent activation/phosphorylation of dimeric STAT transcription factors; the combination of JAK/STAT/SOCS isoforms determining immune gene expression profiles, hence macrophage functionality (reviewed in [92]). In general, STAT1 is associated with IFNγ and IL-12 signaling whereas STAT3 is associated with anti-inflammatory signaling of IL-10 and IL-6. SOCS3 is inducible by IL-10 and IL-6 and can serve to both suppress pro-inflammatory cytokine gene expression as well as negatively feeding back to inhibit IL-10 and IL-6 signaling. *Bifidobacterium* species decreased LPS-induced IL-1 and TNFα mRNA levels in murine RAW264.7 macrophage cells [100], which correlated with inhibition of IκB phosphorylation and increased mRNA levels of SOCS1 and SOCS3. A similar study also demonstrated *B. breve*, LGG and *L. helveticus* to induce macrophage SOCS3 expression [91]. Together, these studies demonstrate that different inflammatory pathways can be modulated by different probiotics in order to induce anti-inflammatory effects. Such anti-inflammatory effects are not exclusively restricted to the direct effect of probiotic-dependent SOCS-suppression of inflammatory cytokines in macrophage cells; LABs inducing the expression of SOCS2 (*L. plantarum*) and SOCS3 (*L. acidophilus*), have been demonstrated to both activate/phosphorylate STAT-1 and STAT-3 whereas inactivating JAK2 and hence, downstream TNFα and IL-8 secretion [90]. Such effects on JAK2 would have profound and discriminatory effects on macrophage polarization and subset-specific responses where JAK2 is essential for GM-CSF and IFNγ signaling but not for IL-6 and IFNα/β signaling (refer to M1 and M2 macrophage subsets later in Section 5.1).

In addition to IL-10, G-CSF has been shown to have anti-inflammatory effects. *L. rhamnosus* strains GG and GR-1 have been shown to elicit the release of G-CSF from macrophages and that G-CSF has a paracrine effect on neighboring macrophages and can suppress inflammatory responses [89]. G-CSF suppresses TNFα production, mediated by the activation of STAT3 and subsequently c-Jun inhibition. Furthermore, strain GR-1 treatment increased G-CSF production in normal human intestinal lamina propria cells. Reduced G-CSF production however, was observed in cells isolated from IBD patient tissue [101]. G-CSF-mediated mechanisms of action are being elucidated; using G-CSFR- deficient DCs, it was observed that GR-1-conditioned media induced significant IL-12/23 p40 production, which indicated that the G-CSF within the GR-1 conditioned media inhibited IL-12/23 p40 production [102]. This suggests that GR-1 can regulate immune responses through releasing G-CSF, and that G-CSF inhibits pro-inflammatory cytokine production via crosstalk between macrophages and dendritic cells. Altogether, these results suggest that production of G-CSF induced by *L. rhamnosus* may have anti-inflammatory effects on key immune cells in the intestine and may be important in maintaining normal immunological homeostasis in the intestine.

Although characterisation of probiotic modulation of MΦ signaling pathways is progressing at a high speed, what is being overlooked however, is the differential effects of probiotics on distinct MΦ subsets. In general, MΦs exist in two canonical subsets; M1 MΦs, which are pro-inflammatory and anti-inflammatory/regulatory M2 MΦs. M2 MΦs are associated with mucosal homeostasis and tolerance, mediated by anti-inflammatory/regulatory cytokines that they produce, including IL-10, TGFβ and IL-1Ra. M1 MΦs, on the other hand, are associated with immune activation and pro-inflammatory responses driven by TNFα, IL-1β, IL-6, IL-8 and IL-12, produced by this subset (reviewed in [110]). The desired effect would be to utilise probiotics to selectively regulate MΦ subsets, whereby M1 MΦs are inhibited in inflammatory pathologies and M2 MΦs inhibited in suppressive pathologies such as mucosal cancers [111]. To this end, a panel of probiotic strains including *L. casei* Shirota, *L. fermentum, L. plantarum, L. salivarius* and *B. breve* have been described to selectively modulate LPS-induced M1 and M2 MΦ production of TNFα, IL-6 and the activation of NFκB; all of which were also dependent on the expression of the TLR2/TLR4-associated co-receptor, CD14 [103,104] (refer to Table 4). The potential of probiotic bacteria to regulate the pro-inflammatory M1 MΦ subset via effects on TNFα secretion and the fact that cytokines often exhibit a level of redundancy have necessitated further investigation to the control of signaling events and secretion of pro-inflammatory cytokines with overlapping functionality to TNFα. Two such cytokines related to each other and exhibiting this functional redundancy to TNFα are IL-1β and IL-18; both of which produced as pro-cytokines and require distinct processing pathways prior to secretion.

### 5.2. Inflammasomes

As a consequence of modulating the expression and secretion of the pro-inflammatory cytokine, IL-1β, probiotics have recently been suggested to play an active role in regulating the expression, assembly and activity of the inflammasome, involved in the caspase-1-dependent processing of both IL-1β and a related pro-inflammatory cytokine, IL-18. The inflammasome is not just involved in processing IL-1β and IL-18 but is also associated with anti-viral responses, through the induction of type I interferons and pyroptosis (inflammatory cell death that exhibits some characteristics of apoptosis) (Reviewed in [112]). This variety of response associated with inflammasome activity is determined by a range of sensor proteins, which associate to form distinct inflammasome subtypes. These inflammasome subtype protein complexes are defined by these sensor proteins, namely NLRP1, NLRP3, NLRP6, NLRC4 (IPAF) and AIM2; each having distinct protein binding partners and sensing either a selective or broad range of activating stimuli. An early investigation has indicated this complexity in inflammasome activity, where two strains of *L. rhamnosus* (LGG and LC705), have been described to process and secrete IL-1β in macrophages. In contrast however, was the differential effect on anti-viral responses; strain LC705 induced the activation of type I interferons whereas LGG did not. This is suggestive of probiotic strain-dependent expression, assembly and activity of distinct inflammasome subtypes in macrophage cells. Thus, probiotic strains may be used to selectively regulate inflammatory responses and anti-viral defences by selectively modulating IL-1β, IL-18 and type I IFNs respectively, through the signaling assembly and activity of distinct inflammasomes.

The NLRP1 inflammasome can be found in monocytes, macrophages, DCs, granulocytes as well as T cells and B cells [113]; this inflammasome can be activated by MDP which results in caspase-1 activation [114] and the further interaction of NOD2 with NLRP1 is desirable for pro-IL-1β expression in an NFκB-independent manner [115]. Further complexity is added to this signaling process by the observation that type I IFNs inhibit NLRP3-mediated caspase-1 activation in a STAT-1-mediated process [116]; such a process may be indicative of a macrophage subset-specific signaling process. STAT1 has been shown to be associated with the pro-inflammatory M1 MΦ subset whereas M2 MΦs utilize STAT3 and STAT6 [110]. This differential utilization of STATs, coupled with responsiveness to cytokines has an impact on the type of inflammasome activated, hence downstream effector functions. As such, the effect of probiotic bacteria on inflammasome isoform, activation and IL-1β/IL-18-mediated functionality can potentially offer an attractive regimen for control of immune cell signaling and warrants extensive investigation.

### 5.3. Modulation of the IL-12/IL-10 Balance

The cytokine response by macrophages varies between different strains of probiotics. Lactobacilli DNA has been identified as a strong inducer of IL-12 production, whereas, bifidobacteria genomic DNA can induce the secretion of the anti-inflammatory cytokine, interleukin-10 [108,117] (refer to Table 4). The mechanisms by which lactobacilli strains differentially induce IL-12 and IL-10 production have been investigated. *L. plantarum* potently induces IL-10 but weakly induces IL-12 production, whereas *L. casei* potently induces IL-12 but weakly induces IL-10 production. It was shown that *L. plantarum* induction of IL-10 was dependent on activation of MAPK pathways, especially ERK; selective blockade of ERK activation resulted in reduced IL-10 production [107]. The key factor for triggering IL-10 production was identified as bacterial teichoic acids, mediated by TLR2-dependent ERK activation. Furthermore, the IL-12-inducing strain, *L. casei*, can be converted into a potent IL-10 inducer in the presence of these teichoic acids. This differential cytokine response was shown to be due to the resistance to intracellular digestion characteristic of lactobacilli strains with a rigid cell wall, such as *L. casei*. Easily digestible lactobacilli, such as *L. plantarm,* or their intact cell walls (ICWs) only weakly induce IL-12 production in macrophages and, furthermore, act as bacteria regulators, inhibiting *L. casei*-induced IL-12 production. However, the ICW of *L. casei* did not inhibit *L. casei*-induced IL-12 production, but its peptidoglycan was sensitive to intracellular digestion and inhibited *L. casei*-induced IL-12 production. This suggests that the suppression of IL-12 by particular lactobacilli is dependent on NOD2, the receptor for the PGN breakdown product, MDP [118]. These studies reveal novel mechanisms for the negative regulation of IL-12 production, whereby easily digestible lactobacilli strains counteract the strong immunostimulatory effects of other lactobacilli strains via NOD2 stimulation and TLR2-dependent ERK activation. This observation demonstrates that selective recognition of specific bacterial components are responsible for the immunoregulatory functions of different probiotic strains and provides theoretical basis for the understanding of their multifunctional activities and thus will contribute to more effective utilization and selection of probiotics in health maintenance and disease prevention.

### 5.4. Lipid Rafts—CD14, TLR2 and TLR4

It is now quite evident that probiotics have the capability to modulate innate immune responsiveness. This modulation may occur by directly suppressing or activating signal transduction molecules, induction of the expression of endogenous negative regulatory molecules, regulation of expression of receptors and their co-receptors or via the induction of exogenous regulatory molecules such as IL-10. One mechanism that is important to innate pattern sensing is the way by which receptors and co-receptors are introduced to form receptor complexes capable of transmitting the reception event. This process depends on the fluidity of the cell membrane and the bringing together of receptor proteins on lipid rafts. Integral to this rafting of related receptor protein complexes is the activity of LysoPC:acyl-CoA acyltransferase-2 (LPCAT2). This enzyme is involved in LPS responses of macrophages and allows for the assembly of CD14 with the LPS receptor, TLR4 as well as the LTA receptor, TLR2 [119]. The activity of LPCAT is up-regulated upon macrophage priming, rendering these cells hyper-responsive to LPS (reviewed in [120]). These priming responses can be driven by the pro-inflammatory cytokines, TNFα and IFNγ [121,122], thus membrane phosphatidyl choline (PC) composition and its effect on membrane fluidity is likely to affect lipid rafting and the association of LPS-sensing receptors and co-receptors [123,124]. Probiotic bacteria have already been implicated in controlling TLR4 expression and TLR4-mediated signaling; it is likely that probiotics may be in future, be implicated in controlling LPCAT2 activity hence phospholipid composition either directly or indirectly through pro-inflammatory priming cytokines such as TNFα and IFNγ. Probiotic modulation of LPCAT2 activity and phospholipid-mediated membrane fluidity potentially offers a way of discriminating between innate immune activation and suppression.

### 5.5. Regulation by miRNA

Micro-RNAs (miRs) are small, non-coding RNAs of approximately 20 nucleotides in length, that regulate cellular responses by binding to target mRNAs and promoting post-translational control of mRNA expression via transcript degradation or inhibition of translation. In general, miRs play an important role in the regulation of inflammation and immune responses through activation or the suppression of innate immunity, through the induction of endotoxin tolerisation (ET). One widely studied micro-RNA, miR-155, has been demonstrated to be a key pro-inflammatory regulator. As such, miR-155 targets known regulators of inflammation including PU.1, SHIP1, SMAD5 and SOCS1 [125]. Genome knock-out of miR-155 in RAW264.7 murine macrophages, up-regulated SHIP1 whereas impaired LPS-induced pro-inflammatory cytokine production (TNFα, IL-6, IL-12) [126,127]. This association of miR-155 with inflammatory response led to a similar investigation, whereby miR-155 was demonstrated to play an important role in macrophage polarisation towards the pro-inflammatory M1 macrophage subset, mediated by LPS and IFNγ. Knock-out of this micro-RNA resulted in the selective suppression of IL-1β and TNFα with no effect on the M2-associated marker enzyme, Arg-1 [128]. Thus, manipulation of miRNAs represents a potential mechanism to selectively regulate inflammatory processes either directly, via modulation of cytokine expression, or indirectly via manipulation of macrophage polarisation and endotoxin tolerisation (ET) mechanisms through negative regulators of PRR signaling. Recent studies have shown miRNA-146a to be up-regulated through TLR2 and further up-regulated upon ET protocols. This TLR2-dependent miRNA-146a induction was shown to suppress TNFα production through a reduction in IRAK-1 expression and IκBα phosphorylation [129]. Given the LTA-TLR2 dependency of certain probiotic modulating mechanisms, the relative expression of miR-146a to miR-155 may represent a future molecular switch, manipulating inflammation versus suppression. In addition, feeding of piglets with *Enterococcus faecium* NCIMB 10415 up-regulated miRNA-423-5p in the small intestine, which was associated with a positive homeostatic effect on immunoglobulin expression, facilitating mucosal barrier functionality [130]. Future studies will further clarify the role of probiotic bacteria in controlling innate cell signaling through the modulation of miRNA expression.

### 5.6. Probiotics as Vehicles of Endotoxin Tolerisation

**Figure 3:**
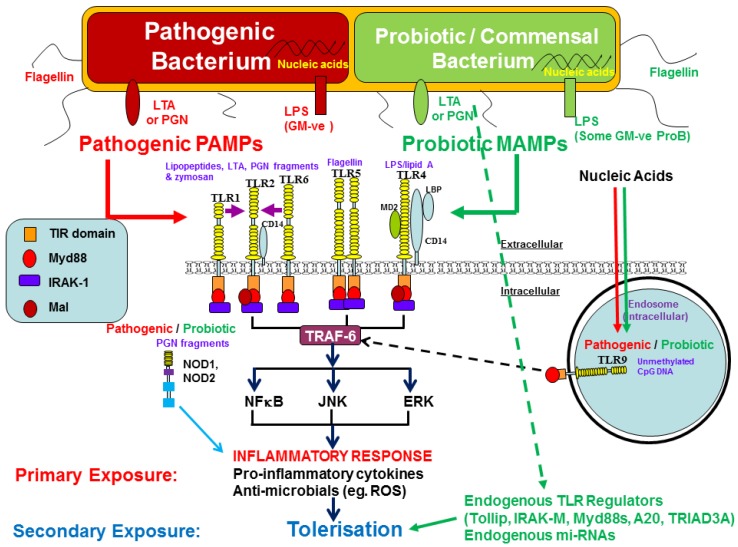
Immune stimulation or tolerisation?—Probiotics hold the key! Probiotics differentially modulate macrophage responses via activation and suppression of distinct pathogen sensing signaling pathways in a strain-dependent manner. Both pathogenic (red) and probiotic (green) bacteria can express similar/overlapping profiles of PAMPs/MAMPs (LTA, LPS, Flagellin, PGN, DNA) through a range of PRRs including external-facing TLR2 (homodimeric or heterodimerised with TLR1 or TLR6), TLR4, TLR5, and intracellular NOD1, NOD2 and endosomal TLR9. All of which can transduce immune activatory/inflammatory responses through activation of NFkB and MAPK signal pathways (indicated in red arrows). In addition, secondary exposure and chronic exposure to probiotic-derived MAMPs (indicated in green arrows), induce a suppressive/tolerogenic response via the induction of endogenous negative regulators to TLR signals (Tollip, IRAK-M, Myd88s, A20, TRIAD3A and miRNAs) that inhibit NFkB and MAPK pathways. Finally, recognition of PGN and its breakdown products through NODs-1 and -2 (indicated in a blue arrow) can have both a positive and negative effect on the inflammatory response that may be determined by selective NOD receptor utilisation or specific structural differences between PAMPs and MAMPs. TIR domain and TLR signal adaptor molecules are indicated in the blue key box.

Probiotic bacteria can be both Gram positive and Gram negative and exhibit a wide range of MAMPs/PAMPs, shared with pathogenic organisms and recognised by PRRs. Thus, probiotic bacteria may initiate responses through a combination of MAMPs; including LTA (TLR2), PGN (NOD-1/NOD-2), DNA (TLR9), Flagellin (TLR5) and LPS (TLR4). The subtleties behind discriminating between initiation of immune inflammatory responses and suppression/regulation of immune responses requires extensive investigation (refer to Figure 3). Such understanding will allow the true harnessing of probiotic and commensal organisms in prophylaxis and direct treatment of a diverse range of immunopathologies. Probiotics are essentially live vehicles for delivery of a variety of MAMPs: simultaneous recognition, staggered or delayed recognition of which will have a profound effect on immune responses. Indeed, staggered recognition of single or multiple different MAMPs is fundamental to hyporesponsiveness in homo- and hetero-tolerisation in innate immune system [reviewed in [131,132].

Understanding the role played by probiotics in driving endotoxin tolerisation (ET) will allow specific and selective utilisation of probiotic bacteria in the treatment of many pathological conditions. The mechanisms by which these organisms tolerise innate immune signaling is diverse. Such mechanisms harnessed in ET have been partially described earlier; these include (1) antagonism of PAMP binding; (2) Down-regulation of PRR expression; (3) Induction of suppressive cytokines e.g., IL-10 [91,105,106,107,108]; (4) Activation of antagonistic pathways e.g., PI3K; (5) Induction of miRNAs [129,130]; (6) Expression of engogenous suppressors of TLR signalling e.g., A20, Tollip, IRAK-M, TRIAD3A and p50/p50 NFκB [91,96,100,109]; (7) Expression and reception events of suppressive receptors e.g., SIGIRR, FcγRIIb (CD32) and Siglecs 3-10 and (8) Cross-regulation of TLR signaling e.g., NOD2 regulation of TLR2, TLR9 regulation of TLR2/4, TLR7/8 regulation of TLR4 ([98] and reviewed in [131]).

Both pathogenic and commensal/probiotic bacteria share common patterns, which activate innate inflammatory signal mechanisms and drive the production of innate activatory cytokines and exogenous signaling molecules. The differential responsiveness to pathogens and probiotics is likely to be determined by subtleties in PAMP/MAMP structure and the relative strength of negative regulatory/tolerisation molecule network. Understanding single, multiple, simultaneous or staggered PRR reception events will clearly unravel the complexities of innate cell activation, differentiation, polarization and tolerisation through probiotic MAMP-PRR signaling, which will facilitate the scientific understanding and potential use of probiotics and their products in the regulation of immune-activatory and suppressive pathologies.

## 6. Secreted Proteins, Lipids, Polysaccharides and Metabolites

With the vast proliferation of investigations that have described immunomodulatory capability of probiotic bacteria is the realisation that the whole organism may not be required, even be replaced by proteins, lipids, polysaccharides and metabolites that are secreted by these bacteria. As such, there are many observations characterising secreted products as immune-modulating and backed up by description of their mechanistic effects on immune cell signaling. There is a vast array of secreted molecules, either fully described or simply as secreted proteins/polysaccharides or conditioned media (refer to Table 5). As early as 2000, extracellular phosphopolysaccharide from *L. delbrueckii* ssp. *bulgaricus* was described to have an immune-enhancing effect on macrophage phagocytosis [133]; although its effect on signaling molecules was not fully appreciated, this study was suggestive of a mechanism which cleared pathogen/cellular debris, hence modulating destructive inflammatory processes.

Several studies have focussed on the effects of secreted products in probiotic conditioned medium. Purified proteins from LGG conditioned media (p75 and p40) have been shown to activate PKB/Akt involved in the suppression of TNFα-mediated epithelial cell damage [134]. Conditioned media from *L. plantarum* (Lp-CM) inhibited TNFα-induced MCP-1 secretion by epithelial cells and macrophages via inhibition of NFκB and proteasome activity [135]. Supernatant from culture of *L. fermentum* inhibits *Y. enterocolitica*-induced epithelial cell IL-8 by suppressing Rac, p38 MAPK and NFκB activity. This suppressive activity being abrogated by phospholipase C, indicating the suppressive activity to be mediated by a secreted phospholipid [136]. In addition, *L. rhamnosus* L34 and *L. casei* L39 conditioned media suppress *C.difficile*-induced colonic epithelial cell IL-8 and GM-CSF production: L34 suppressed NFκB whereas L39 suppressed both NFκB and c-jun phosphorylation [81]. Thus, conditioned media from probiotic bacterial species has been demonstrated to either activate (PKB/Akt) or suppress (Rac, MAPKs, NFκB) a variety of signaling pathways that is both strain-dependent and dependent on the nature of the secreted products (proteins and lipids). Early mass-spectrometry data is suggestive that *L. rhamnosus* GG secretes Serpin β_1_ and a transcriptional regulator [137]. Employment of further proteomic and lipidomic approaches will reinforce our understanding of secreted products capable of modulating innate immune signaling.

As a result of gastrointestinal processing of probiotic bacteria, products, such as DNA and cell wall components, released upon breakdown of probiotics may also have a profound effect on innate signaling. Indeed, DNA from bifidobacteria has been described to activate macrophage expression of NO, IL-1β, IL-6, IL-12p40 and TNFα), thus driving an activatory/pro-inflammatory response [108,139]. In comparison, purified cell wall from *L. gasseri* also stimulates an inflammatory response, inducing the expression and secretion of IL-1β, TNFα, MIP-1α, MCP-1, IL-10 and NO [82]. Whereas pLTA derived from the cell wall of *L. plantarum* antagonises and cross-tolerises LTA and PGN-induced inflammatory responses via suppression of NFκB and MAPK signaling pathways [96,97,98].

Finally, anaerobic metabolic breakdown of complex carbohydrates can result in the production of products such as the short chain fatty acids, butyrate, proprionate and acetate. Indeed, butyrate, which can also act as a histone deacetylase inhibitor, has been shown to inhibit pro-inflammatory cytokine expression in both monocytes and macrophages while simultaneously inducing the expression of the anti-inflammatory cytokine, IL-10 [105]. This has been suggested via a mechanism involving the inhibition of NFκB activation [140]; this is not the case however, in the study using THP-1-derived macrophages [105], where it is possible that the inhibitory effect on pro-inflammatory cytokines may be downstream, and secondary to a positive regulation of IL-10 or indeed to butyrate modulation of NOD2 responsiveness to PGN [141]. Manipulation of responsiveness to butyrate and expression of receptors to SCFAs offer another mechanism for control of inflammatory mechanisms.

## 7. Conclusions

Probiotics have been used to treat a range of health conditions encompassing intestinal and extraintestinal sites, including atopic dermatitis, necrotising enterocolitis, pouchitis and ulcerative colitis [142,143,144]. The mechanisms for the observed health effects of these probiotics are not fully understood, but are likely due to the direct/indirect action on the intestinal immune system. As summarized in this review, probiotics have been documented to modulate key immune responses within the gut by modulating signaling pathways including NFκB, MAPK, PI3K/Akt and JAK/STAT signaling. The actions of these probiotics are seen within IECs, DC, neutrophils and macrophages, however the probiotic mechanisms of action are often strain-specific and can have varied responses within different host cells. If the use of probiotics is to continue, it will be of crucial importance to identify the precise probiotic responses and relevant effector molecules, along with the effected host cell signaling events. This will allow us to explore the effects of different strains on host immune responses and elucidate currently unknown probiotic modulation of signaling events within host cells.

Future directions should continue to study gene expression to explore how bacteria affect intestinal biology and to elucidate cellular populations within the intestinal microbiota to identify how these contribute to changes in intestinal physiology. This review has identified the diversity of effector functions produced by different strains of the same bacterial species. In combination with functional and comparative gene-based approaches, a greater understanding of strain-specific effector functions and the ways in which such strains modulate signaling events, will enable selection of the most appropriate probiotic strain or possible genetically-engineered probiotic to treat gastrointestinal diseases.

There are many areas of probiotic-immunomodulation, that require extensive investigation, one of which is the role of probiotics in modulation of APC function, in particular macrophages. These APCs are able to regulate local immune responses by activating or suppressing inflammatory pathologies. In particular, mucosal macrophages have been characterised into effector subsets, M1 (pro-inflammatory) and M2 (anti-inflammatory/regulatory). Manipulation of macrophage plasticity by probiotics through regulation of effector functions via manipulation of intracellular signaling pathways may hold potential for macrophage-based therapies to treat mucosal inflammatory pathologies.

Given that certain probiotics have already shown to be a successful regime for particular health conditions, future development should therefore focus on underpinning the specific signaling mechanisms of immunopathologies within the gut and how they may be specifically, and selectively modulated by probiotics. As indicated in this review, probiotics can exert modulatory effects on signal pathways involved in immune activation, deviation or regulation/suppression, therefore probiotic strain selection or probiotic combination should be carefully considered, in the context of immune cell signaling, in order to achieve the desired immunomodulatory effect.

## Figures and Tables

**Table 1:** Probiotic modulation of signaling pathways in intestinal epithelial cells.

Probiotic Species (Soluble Product)	Probiotic–Induced Effector Response	Cells/Cell Line	Signaling Pathway	Reference
*Bifidobacteria breve* M-16V *Bifidobacteria longum* BB536	Increase IL-8, MCP-1 and IL-6 levels	PIE cells	NFκB via activation of A20	[25]
*Bifidobacteria lactis* KCTC 5727	Suppress NFκB-binding activity & IκB degradation	HT-29 cells	NFκB	[26]
*Lactobacillus amylovorus* DSM 16698T	Decrease ETEC-induced IL-8 & IL-1β production	Caco-2/TC7 cells (higher transport activity).	TLR4 signaling via activation of Tollip and IRAK-M	[27]
*Lactobacillus casei* OLL2768	Decrease IL-6, IL-8, IL-1α and MCP-1	BIE cells	NFκB and p38 MAPK	[28]
*Lactobacillus rhamnosus GG*	Increased expression of IL-10R2	*Ex vivo* C57 BL/6J mice Immature colon samples	Phospho-STAT3, increased expression of SOCS-3	[29]
*Lactobacillus rhamnosus GG*	TLR2 up-regulation	IPEC-J2	TLR signaling	[18]
*Lactobacillus rhamnosus GG* (LGG)	Reduce NFκB activation via ROS	FHs74Int cells (human foetal)	NFκB	[30]
*Lactobacillus rhamnosus GG* p40 and p75 proteins	Enhance tight junction	YAMC	EGFR/Akt	[23,24,31]
*Lactobacillus rhamnosus GG p40 protein* (LGGp40)	Decrease cytokine-induced apoptosis	YAMC	EGFR/Akt via Src	[22]
*Lactobacillus jensenii* TL2937	Activate negative regulators A20, Bcl-3 and MKP-1	PIE cells	TLR4-dependent NFκB and MAPK	[32]
*Lactobacillus plantarum* (LP) WCFS1	Increased tight junction proteins	Caco-2 cells	TLR2 signaling	[17]
*Lactobacillus reuteri* DSM 17938	Inhibit IκB phosphorylation	*Ex vivo* rat model	NFκB	[33]
*Saccharomyces cerevisiae* (Sc) CNCM I-3856	Decrease IL-6 and IL-8 expression	IPEC-1	Decrease of ERK1/2 and p38 phosphorylation	[34]
*Streptococcus salivarius* K12	Inhibit NFκB activity	HT-29	NFκB	[35]
VSL#3	Increased tight junction proteins	HT-29 cells	Phosphorylation of ERK and p38 MAPKs	[20]
VSL#3	Induction of heat shock proteins (hsp)	Colonic IECs	Suppression of NFκB via inhibition of proteosome	[36]

Probiotics differentially modulate epithelial cell responses via activation or suppression of distinct signaling pathways in a strain-dependent manner. Observations presented include a range of strains of bifidobacteria, lactobacilli, streptococcus, VSL#3 mixture and the yeast, *S. cerevisiae* (*Sc*). Epithelial cell models include human (Caco-2, Caco-2/TC7 (late passage clone), HT-29, FHs74Int), murine (YAMC), porcine (porcine intestinal epithelium PIE, intestinal porcine epithelial cells, IPEC), and bovine (BIE) cells. Endogenous negative regulators of TLR signaling (IRAK-M, Interleukin receptor-associated kinase-M; Tollip, Toll interacting protein; A20, TNF-inducible zinc finger protein A20; Bcl-3, proto-oncogene 3 of B cell chronic lymphocytic leukaemia; MKP-1, mitogen-activated protein kinase phosphatase-1). Cytokines indicated are Interleukin (IL)-1α, -1β, -6, -8, -10, IL-10R2, IL-10 Receptor subunit 2 and MCP-1, monocyte chemoattractant protein -1. ROS, reactive oxygen species. NFκB, Nuclear factor kappa B; ETEC, Enterotoxigenic *Escherichia coli*; TLR, Toll-Like Receptor; MAPK, mitogen activated protein kinases; SOCS, suppressor of cytokine signaling; EGFR, epithelial growth factor receptor; ERK, extracellular signal-related kinases.

**Table 2:** Probiotic modulation of signaling pathways in intestinal dendritic cells.

Probiotic	T Cell Activation	PRR and Signaling Pathway	Reference
*Bifidobacterium breve* Yakult strain	Tr_1_ cells	TLR2/MyD88	[60]
*B. longum* BB536 DNA	Increased CD4^+^CD25^+^ Treg cells & Th_1_ activation	TLR9 and IκB-α phosphorylation	[55]
*IRT5*	CD4^+^Foxp3^+^ regulatory T cells	Not described.	[62]
*LGG* DNA	Increased CD4^+^CD25^+^ Treg cells and Th_1_ activation	TLR9 and IκB-α phosphorylation	[55]
*LGG* DNA	ND	TLR9 and reduced IκBα degradation and p38 phosphorylation	[56]
*Lactobacillus paracasei* ATCC 25302 DNA	CD4^+^ Foxp3^+^ Treg cells	TLR9	[57]
*Lactobacillus salivarius* Ls33	CD4^+^ Foxp3^+^ Treg cells	NOD2	[61]

Probiotics differentially modulate dendritic cell responses via activation of distinct pathogen sensing signaling pathways (TLR2, TLR9, NOD2) in a strain-dependent manner. Table also indicates the downstream effects on effector T cells (Th_1_) and regulatory T cells (Treg). Observations presented include a range of strains of Bifidobacterium, lactobacilli, and IRT5 probiotic mixture and the modulatory effect of probiotic DNA from *L. rhamnosus* GG and *B. longum* BB536. ND, indicates not determined.

**Table 3:** Probiotic modulation of neutrophil effector function and signaling pathways.

Probiotic	Effector Response	Cells/Cell Line/Model	Reference
*L. gasseri* NC1500 *L. gasseri* + MnSOD NC1501	Reduction in severity of inflammation —decreased Nφ & Mφ infiltration	IL-10 deficient mouse model of colitis	[75]
*B. animalis* MB5*L. rhamnosus* GG Probiotics & Secreted factors	Inhibition of Nφ transmigration by suppression of IL-8, Gro-α, ENAP-78 and suppression of chemokine regulators, IL-1β & TNFα.	Enterotoxigenic *E.coli* K88 – induced inflammation in Caco-2 epithelial cells	[76]
*B. lactis* HN019	Increased Nφ phagocytic capacity & enhanced phagocyte-mediated bacteriocidal activity.	Clinical trial: Healthy human PBMCs	[77]
*E. coli* Nissle 1917	Amelioration of inflammation: Reduced levels of Nφs & chemokines.	DSS-induced colitis, murine model. Prophylaxis – faecal transplantation model.	[78]
*L. rhamnosus* NutRes1 *B. breve* NutRes204	Worsening of faecal condition accompanied by reduced Nφ numbers. *L. rhamnosus* improved this & increased TLR2/6, CCL2, TNFα, IL-1β, IL-6.	DSS-induced chronic colitis, murine model. Relapse–Remision cycling.	[79]
*B. longum* subsp. Infantis BB-02	Reduction in inflammation: decreased Nφ infiltration & CXCL-1.	DSS-induced acute colitis, murine model.	[80]
*L. rhamnosus* L34 *L. casei* L39	Suppression of IL-8 expression. Conditioned media: L34 suppressed NFκB phosphorylation, L39 suppressed both NFκB & c-Jun.	*C. difficile*-associated disease colitis model. Infant faecal isolates on HT-29 epithelial cells.	[81]
*L. gasseri* ATC33323 cell wall extract	Cell wall extract—lethality. Increased TNFα, IL-1β, MIP-1α, MCP-1, NO & decreased Nφ count.	Sepsis model in Sprague-Dawley rats.	[82]
*L. rhamnosus* Lcr35	AM development prevented by suppressing IL-4 (Th2), IL-17 (Th17), TSLP via a Foxp3^+^ Treg-dependent mechanism.	Murine OVA challenge allergic march (AM) model.	[83]
*L. rhamnosus* GG	Inhibition of PMA- and *S.aureus*-induced neutrophil extracellular traps (NETs), ROS production & phagocytic capacity.	HL60-derived Nφ cell model.	[84]
*B. longum* OLL6001 Culture condensate (BCC)	Augmentation of Nφ recruitment. Upregulation of adhesion molecule & cytokine expression. Immuno-enhancing.	Diet-restricted murine peritonitis model: ip glycogen injection.	[85]
VSL#3 Probiotic mixture	Reduction in mucosal levels of Nφ chemoattractant, IL-8 & tissue influx of Nφs.	Human retrospective study of proB-treated UC pouchitis.	[86]
*L. plantarum* ATCC10241	Reduction in bacterial load, Nφ numbers, apoptotic/necrotic cells and IL-8. Wound healing.	Chronic infected leg ulcers (diabetic and non-diabetic).	[87]
*Clostridium butyricum* MIYAIRI 588	Anti-tumour effects by MMP-8 driven release of TRAIL (TLR2/4–dependent)	Nφs in BCG-responsive bladder cancer patients	[88]

Probiotics differentially modulate neutrophil effector responses via activation of distinct pathogen sensing signaling pathways in a strain-dependent manner. Observations presented include a range of LAB strains of bifidobacteria, lactobacilli, and *Escherichia coli* Nissle 1917. Effector responses to probiotic introduction are indicated as modulation of phagocytosis, killing activity, inflammation and cytokine production, which by inference are linked to effects on pathogen sensing and signaling pathways. These observations have been recorded for a range of neutrophil studies using distinct primary cells, cell lines and in vivo models indicated.

**Table 4:** Probiotic modulation of signaling pathways in macrophage cells.

Probiotic	Effector Response	Cells/Cell Line	Reference
*L. casei* Shirota *L. fermentum* MS15 *L. plantarumNCIMB41605 L. salivarius NCIMB41606 B. breve* NCIMB 8807	TNFα: Increased—CD14^hi^ M1/M2 Decreased—CD14^lo^ M1/M2 IL-6: Decreased—M2 NF-κB: Decreased—M1 Increased—M2	M1 & M2 Mφ subsets (THP-1 cell line)	[103] [104]
Secreted Protein	+/− IL-6 M1 Mφs +/− IL-8/TNFα	M1 & M2 Mφ subsets (THP-1)	[103] [104]
SCFA-butyrate	Suppression of IL-12 & augmentation of IL-10	Human monocytes	[105]
SCFA-butyrate	Decreased TNFα Increased IL-10	M1 & M2 Mφ subsets (THP-1)	[106]
*L. helveticus DSM13137 L. rhamnosus* GG *S. thermophilus* THS *B. breve* DSM13692	Increased IL-10:IL-12 ratios Induced SOCS3 (IL-10 & p38 MAPK-dependent)	Human Mφs GM-CSF-PB monocytes	[91]
*L. plantarum* K8 KCTC10887BP LTA (pLTA)	Suppression of LPS Induced TNFα (pLTA supp^n^ of ERK, JNK, p38 MAPK phosphorylation, IκB degradation & TLR4. Induction of IRAK-M.	THP-1 & U937 pro-monocytes Murine sepsis model L929-BMM Balb/c	[96]
*L. plantarum* KCTC10887BP LTA (pLTA)	Suppression of *S. aureus* LTA (aLTA) induced TNFα pLTA suppresses Myd88, NFκB & MAPKs. Antagonises aLTA.	THP-1 pro-monocytes	[97]
*L. plantarum* KCTC10887BP LTA (pLTA)	Suppression of *S. flexneri* PGN Induced inflammation (TNFα, IL-1β). Tol^n^ decreases NOD2; pLTA cross Tol^n^ Flex PGN	THP-1 pro-monocytes	[98]
*B. breve* ATCC15700 *B. longum* ATCC15697 *Enterococcus faecalis* ATCC19433	Differential modulation of TNFα, IL-1β, IL-12p40 mRNA. Decreased IκB phosphorylation and induction of SOCS-1, -3.	RAW264.7 murine Mφs LPS-stimulated	[100]
LAB LTA *L. plantarum* ATCC14917^T^ *L.casei* YIT9029	Regulate TLR2-dependent ERK mediated IL-12/IL-10 axis: LP high IL-10/IL-12, LC low IL-10/IL-12.	Murine peritoneal Mφs Balb/c	[107]
*L. rhamnosus* GR1 & GG	G-CSF-mediated inhibition of JNK: Suppression of TNFα	Murine immort peritoneal & BMM C57Bl/6 Human THP-1s	[89]
ProB DNA *L. casei* & *B.breve*	Induction of IL-1, IL-10 & IL-6	Human PBMCs	[108]
*L. paracasei* Cultech	TLR2-dependent up-regulation of negative regulators of NFκB	Human PBMCs & PMA-THP-1 cells.	[109]
*L. reuteri* ATCC PTA 6475 CF48-3A & ATCC55730 conditioned medium	Suppression of LPS-induced TNFα & MCP-1 via inhibition of MAPK-driven cJun/AP-1act^n^	THP-1 cells, MonoMac-6 cells, CD Mφs	[99]

Probiotics differentially modulate macrophage responses via activation of distinct pathogen sensing signaling pathways in a strain-dependent manner. Observations presented include a range of strains of bifidobacteria, lactobacilli, and streptococci and enterococci and the modulatory effect of probiotic DNA, conditioned medium, cell wall lipoteichoic acid (LTA) and the short-chain fatty acid (SCFA) metabolite, butyrate. Effector responses to probiotic introduction (and their products) are indicated as modulation of inflammation, pathogen sensing, cytokine production and their linkage to effects on signaling molecules and pathways. These observations have been recorded for a range of macrophage studies using distinct primary cells, cell lines and ex vivo models indicated.

**Table 5:** Probiotic-derived MAMPs, secreted products and metabolites modulate immune signaling pathways.

Probiotic Format	Effector Response	Cells/Cell Line	Reference
*L. fermentum* MS15 *L. plantarum NCIMB41605 L.Salivarius* NCIMB41606 *B.Breve* NCIMB8807 Secreted Protein	TNFα: Increased—CD14^hi^ M1 Selective suppression—CD14^lo^M1/M2 IL-6: Decreased—M2 NFκB: Selective suppression—CD14^hi^M1. Increased—M2	M1 & M2 Mφ subsets (THP-1 cell line)	[103]
*L. casei* Shirota Secreted Protein	Suppressed LPS-induced TNFα, NFκB activation & augmented IL-1β (M1 Mφs). Suppressed LPS-induced IL-6 & augmented IL-1β (M2 Mφs), independent of NFκB activation.	M1 & M2 Mφ subsets (THP-1 cell line)	[104]
SCFA-butyrate	Suppression of LPS/PGN-induced TNFα (M1 & M2). Suppression of LPS/PGN-induced IL-1β (M2). Augmentation of LPS/PGN-induced IL-10 (M2). Suppression of monocyte LPS/PGN—induced TNFα & IL-1β.	M1 & M2 Mφ subsets (THP-1 cell line)	[106]
*L. plantarum* (Non-designated strain) Conditioned medium (Lp-CM)	Inhibition of NFκB binding activity andproteosome-dependent degredation of IκBα. (Stimuli: TNFα, LPS, Flagellin, Poly I:C). Suppression of MCP-1 secretion.	Murine YAMC intestinal epithelial cells, RAW264.7 Mφs, Primary DCs	[135]
*L. plantarum* K8 KCTC10887BP LTA (pLTA)	Suppression of LPS-induced TNFα pLTA supp^n^ of ERK, JNK, p38 MAPK phosphorylation, IκB degradation & TLR4. Induction of IRAK-M expression.	THP-1 & U937 pro-monocytes. Murine sepsis model L929-BMM Balb/c	[96]
*L. plantarum* KCTC10887BP LTA (pLTA)	Suppression of *S. aureus* LTA (aLTA) induced TNFα pLTA suppresses Myd88, NFκB & MAPKs. Antagonises aLTA.	THP-1 pro-monocytes	[97]
*L. plantarum* KCTC10887BP LTA (pLTA)	Suppression of *S.flexneri* PGN induced inflammation (TNFα, IL-1β). Tol^n^ decreases NOD2; pLTA cross Tol^n^ Flex PGN	THP-1 pro-monocytes	[98]
*L. rhamnosus* GG p75 & p40 secreted proteins	Protection from H_2_O_2_-mediated damage to IEC barrier TJs (PKC & MAPK-dependent).	IECs	[134] [138]
LAB LTA *L.plantarum* ATCC14917^T^ *L.casei* YIT9029	Regulate TLR2-dependent ERK mediated IL-12/IL-10 axis: LP high IL-10/IL-12, LC low IL-10/IL-12.	Murine peritoneal Mφs Balb/c	[107]
*L. rhamnosus* GG secreted protein Serpin B1, protease inhibitor of neutrophil elastase. Band G2 & G4.	Prevention of epithelial cell barrier damage.	Mass Spec. (MS/MS) analysis of LGG S/N grown in MRS broth.	[137]
ProB DNA *L.casei* & *B.breve*	Induction of IL-1, IL-10 & IL-6	Human PBMCs	[108]
*L. rhamnosus* GG DNA	Immunostimulation	Murine immune cells B cells	[117]
Bifidobacteria (Non-designated strain) unmethylated CpG DNA	Augmentation of Mφ phagocytosis, NO release and secretion of IL-1β, IL-6, IL-12p40 and TNFα (CpG DNA recognised by TLR9).	Murine Mφs : J774A.1 cells	[139]

Probiotic-derived products differentially modulate immune cell effector responses via activation of distinct pathogen sensing signaling pathways. Observations presented include the modulatory effect of probiotic DNA, conditioned medium, cell wall lipoteichoic acid (LTA) and the short-chain fatty acid (SCFA) metabolite, butyrate for a range of strains of bifidobacteria and lactobacilli. Effector responses to introduction of probiotic-derived products are indicated as modulation of inflammation, pathogen sensing, cytokine production and their linkage to effects on signaling molecules and pathways. These observations have been recorded for a range of studies using distinct primary cells, cell lines and ex vivo models indicated.
